# Therapeutic Drug Monitoring of Antifungal Agents in Critically Ill Patients: Is There a Need for Dose Optimisation?

**DOI:** 10.3390/antibiotics11050645

**Published:** 2022-05-12

**Authors:** Daniela Baracaldo-Santamaría, Juan David Cala-Garcia, Germán José Medina-Rincón, Luis Carlos Rojas-Rodriguez, Carlos-Alberto Calderon-Ospina

**Affiliations:** 1 Pharmacology Unit, Department of Biomedical Sciences, School of Medicine and Health Sciences, Universidad del Rosario, Bogotá 111221, Colombia; daniela.baracaldo@urosario.edu.co (D.B.-S.); germanj.medina@urosario.edu.co (G.J.M.-R.); luisca.rojas@urosario.edu.co (L.C.R.-R.); 2 Pulmonary Care at Baylor College of Medicine, Houston, 77030 TX, USA; juand.cala@urosario.edu.co; 3 GENIUROS Research Group, Center for Research in Genetics and Genomics (CIGGUR), School of Medicine and Health Sciences, Universidad del Rosario, Bogotá 111221, Colombia

**Keywords:** drug monitoring, antifungal agents, invasive fungal infections, echinocandins, azoles, amphotericin B, pharmacokinetics, pharmacology

## Abstract

Invasive fungal infections are an important cause of morbidity and mortality, especially in critically ill patients. Increasing resistance rates and inadequate antifungal exposure have been documented in these patients, due to clinically relevant pharmacokinetic (PK) and pharmacodynamic (PD) alterations, leading to treatment failure. Physiological changes such as third spacing (movement of fluid from the intravascular compartment to the interstitial space), hypoalbuminemia, renal failure and hepatic failure, as well as common interventions in the intensive care unit, such as renal replacement therapy and extracorporeal membrane oxygenation, can lead to these PK and PD alterations. Consequently, a therapeutic target concentration that may be useful for one patient may not be appropriate for another. Regular doses do not take into account the important PK variations in the critically ill, and the need to select an effective dose while minimising toxicity advocates for the use of therapeutic drug monitoring (TDM). This review aims to describe the current evidence regarding optimal PK/PD indices associated with the clinical efficacy of the most commonly used antifungal agents in critically ill patients (azoles, echinocandins, lipid complexes of amphotericin B, and flucytosine), provide a comprehensive understanding of the factors affecting the PK of each agent, document the PK parameters of critically ill patients compared to healthy volunteers, and, finally, make recommendations for therapeutic drug monitoring (TDM) of antifungals in critically ill patients.

## 1. Introduction

Infection is an important cause of morbidity and mortality in intensive care units (ICUs) around the world. The majority of microbiological cultures taken from critically ill patients suffering from infectious diseases are caused by gram-negative and gram-positive organisms, but an estimated 19% of the positive isolates are fungi, most commonly *Candida* and *Aspergillus* [1]. The frequency of fungal infections in critically ill patients is increasing [2] even in non-immunocompromised hosts [3], which is concerning given the high mortality rates of invasive fungal infections (IFIs). For instance, it has been estimated that invasive candidiasis is diagnosed at a rate of 2.6 cases per 100 ICU admissions, with a mortality rate of 58.6%, and with increasing resistance rates to fluconazole (in 27.9% of cases), caspofungin (2.9%) and amphotericin B (3.1%) [4]. Furthermore, patients are at risk of systemic fungal infections following solid organ transplantation, where up to 68% of patients who develop invasive pulmonary aspergillosis die [5]. In addition, fungal coinfections in patients with COVID-19 also have high mortality rates. Bretagne et al. found a mortality rate ranging from 36.6% to 76.7% for COVID-19-associated pulmonary aspergillosis [6], and interestingly, some studies have found that for patients with candidemia, one of the risk factors for mortality is inadequate antifungal therapy [7]. This leads us to question whether the dose regimen currently used is adequate for all patients, including the critically ill.

The treatment of IFIs is often difficult due to emerging resistance and the need for early therapeutic intervention, and therefore appropriate choice and dosing of antifungal agents is crucial in determining clinical outcome [4,8]. Antifungal agents commonly administered for systemic use in critically ill patients are divided into four classes: triazoles, echinocandins, polyenes and fluoro-pyrimidines. All of them show clinically relevant pharmacokinetic (PK) and pharmacodynamic (PD) alterations in critical states, which could explain decreased drug exposure in critically ill patients, leading to treatment failure. The PK of antifungal drugs can vary due to a number of physiological changes including third spacing, hypoalbuminemia, renal failure or hepatic failure, as well as common interventions in the ICU such as renal replacement therapy and extracorporeal membrane oxygenation [9,10]. Furthermore, many patients in the ICU are in a septic state, in which the endothelial dysfunction secondary to inflammation seen both in sepsis and septic shock leads to capillary leakage that in turn causes an expansion of the interstitial space and can increase a drug’s volume of distribution (Vd), especially for hydrosoluble compounds [11]. Hypoalbuminemia seen in critically ill patients may also contribute to increased Vd and significantly affect agents that have high protein-binding capacity (e.g., echinocandins), leading to an increased amount of unbound drug, which itself results in greater distribution [12].

Due to the high mortality rates of IFIs in the critically ill, and the emerging resistance of *Candida* isolates [13,14], there is a need to optimise the dose of antifungal pharmacotherapy, because a therapeutic target that may be useful for one patient may not be appropriate for another. Regular doses do not take into account important PK variations in the critically ill [15], and the selection of an effective dose while minimising toxicity advocates for the use of therapeutic drug monitoring (TDM). The objective of TDM is to ensure target exposure is being attained, so that the treatment has an impact on fungal growth and consequently on clinical outcomes [16].

The use of echinocandins in the ICU has been reported to show pronounced inter-individual and inter-occasion variability in clearance (Cl) and therefore drug exposure in the critically ill, compared to healthy patients. For echinocandins, Kapralos et al. found an estimated inter-individual variability in Cl of 45.1% and an inter-occasion variability in Cl of 31%. Furthermore, they revealed that the current dose regimen had a low percentage of target attainment (PTA) for *C. albicans*, *C. glabrata*, and *C. parapsilosis* with a minimal inhibitory concentration (MIC) of ≥0.06 mg/L, ≥0.12 mg/L, and 0.12 mg/L respectively [17]. In addition, altered PK of azoles in critically ill patients has also been documented, and was well illustrated in the DALI (edfining antibiotic levels in the intensive care unit) study, in which 33% of patients treated with fluconazole did not achieve the optimal PK/PD index (fAUC_0–24_/MIC ≥ 100) (the ratio of the area under the concentration-time curve to the MIC) [18].

For a drug to be considered a candidate for TDM it must show certain characteristics. For example, the drug must demonstrate a relationship between systemic concentrations and efficacy or toxicity, must ensure that the recognition of the plasma concentration would aid in clinical decision-making and lead to dose adjustment, and the drug should also demonstrate large inter-patient variability or have a narrow therapeutic range [16]. Establishing a PK/PD index for each antifungal agent would help to correlate drug exposure with efficacy, and should be considered essential to improve patient outcomes and to reduce the emergence of resistant pathogens. This review aims to describe the current evidence regarding optimal PK/PD indices associated with the clinical efficacy of the most commonly used antifungal agents in critically ill patients (azoles, echinocandins, lipid complexes of amphotericin B, and flucytosine), provide a comprehensive understanding of the factors affecting the PK of each agent, document the PK parameters of critically ill patients compared to healthy volunteers, and discuss the strategies supporting TDM of antifungals in critically ill patients.

## 2. TDM of Azoles in Critically Ill Patients

The antifungal group of azoles is associated with two subgroups: imidazoles and triazoles; the first is formed by a two-nitrogen azole ring and the second is formed by a three-nitrogen ring [19]. Both subgroups work by inhibiting the cytochrome P450 enzyme 14-a-sterol-demethylase, a key enzyme in the synthesis of fungal ergosterol [20], which is the most abundant sterol in fungi membranes and the main actor in maintaining membranes’ permeability and fluidity [21]. The main protagonists of this subgroup of antifungals in current clinical practice are fluconazole, voriconazole, posaconazole, isavuconazole and itraconazole [19]. Of these antifungals, only itraconazole, posaconazole and voriconazole might be candidates for TDM, primarily due to increased risk of serious adverse reactions and drug-drug interactions [22].

### 2.1. Itraconazole

Itraconazole has a broad-spectrum efficacy against multiple fungi such as *Blastomyces* spp., *Histoplasma*, *Coccidioides*, *Aspergillus* and many dermatophytes [22]. Despite its great coverage, itraconazole should be used carefully due to its unpredictable oral bioavailability, as well as the many significant drug-to-drug interactions it might have [23]. Itraconazole can be administered as oral capsules or IV (intravenous) preparations. Table 1 shows the available evidence of pharmacokinetic parameters for itraconazole in healthy volunteers and critically ill patients using different formulations. According to the British Society for Medical Mycology, standard IV itraconazole dosage in the adult population is a loading dose of 400 mg for 2 days followed by 200 mg/day, while for oral formulation, 200 mg/day can be formulated in 1–2 doses for treatment against esophageal candidiasis with pathogens showing decreased susceptibility to fluconazole [24]. Despite its fixed dosage, itraconazole should be administered carefully due to its non-linear pharmacokinetics [25]. In a critical patient setting, Vandewoude et al. demonstrated that a seven-day IV scheme followed by twice-daily oral administration for two weeks showed adequate itraconazole and hydroxy-itraconazole (an active metabolite which also has antifungal properties) plasma levels, reaching a steady state in the 96th hour of the IV protocol and obtaining an average plasma level of 550 ng/mL, which is above the necessary MIC (0.250 to 0.500 mg/mL) [26]. Itraconazole levels should be measured on the 5th−7th day, aiming for concentrations >0.5 mg/L for both prophylactic and therapeutic indications [24]. Lower levels have been associated with higher mortality [27], and better outcomes regarding infections with *Aspergillus*, *Cryptococcus neoformans* or *Histoplasma capsulatum* have been associated with higher itraconazole plasma concentrations [28,29,30,31]. These findings are congruent with the PK drug exposure of itraconazole, in which C_min_ is used rather than AUC to evaluate the itraconazole exposure target [32]. A C_min_ range of 0.5–1 mg/L is considered the optimal level for itraconazole treatment [32]. Adverse reactions have been documented with higher concentration levels [33]. Lestner et al. suggested that levels above 5 mg/L increase the probability of an adverse event by 26% [34].

TDM of itraconazole also results in a decrease in adverse reactions, problems that are not uncommon with this drug. Common side effects are nausea, vomiting, elevation of hepatic enzymes or hypokalemia. Less common side effects that have significant clinical impacts are cardiovascular pathologies such as acute heart failure, hypertension, premature ventricular contractions and, less commonly, ventricular fibrillation [35]. Its usage is also contraindicated in pregnancy and lactation [19]. Interactions between itraconazole and other drugs should be carefully reviewed due to the strong inhibition of cytochrome P450 (more specifically CYP34A) [25]. Coadministration of carbamazepine, phenytoin or rifampin could decrease itraconazole’s therapeutic effect, while simultaneous administration of itraconazole with warfarin or cyclosporine could increase the therapeutic effect of the drugs mentioned previously [25].

**Table 1:** Summary of Clinical Studies Assessing PK Parameters of Itraconazole.

Clinical Context	Dose	AUC_0–24_ (mg × h/L)	C_min_ (mg/L)	C_max_ (mg/L)	Cl (L/h)	Vd (L)	Reference
10 ICU patients with IFIs	IV formulation 200 × 2 days 1 & 2, followed by 200 mg daily	29.3 ± 6	0.37 ± 0.17	1.2 ± 0.3	-	-	[36]
Healthy volunteers	200 mg capsules (with food)	45.2 ± 10.8	1.86 ± 0.54	2.23 ± 0.51	-	-	[37]
Healthy volunteers	200 mg capsules once daily (with food)	15.4 ± 6.9	0.42 ± 0.18	1.07 ± 0.05			[38]

### 2.2. Posaconazole

Posaconazole is a broad-spectrum antimycotic which has a very similar chemical structure to itraconazole. Posaconazole has demonstrated good activity against *Candida*, *Cryptococcus*, *Aspergillus* and *Mucoraceous* species [24]. Currently its formulations are IV solution, oral solution or capsules. The oral formulations have variable bioavailability, although in the adult population, capsules might have a bioavailability greater than 70–80% [39]. The pharmacokinetics of the available clinical studies of this drug are described in Table 2. In terms of TDM, the main parameter identified to monitor its action is the AUC/MIC ratio [40,41], which predicts better eradication of yeasts in in vivo experiments [40]. For clinical purposes, target plasmatic concentrations of >0.7 mg/L in prophylaxis (or >1–1.25 mg/L in treatment of invasive fungal disease) [39] correlate with better clinical outcomes, and these levels should be measured within one week from starting therapy [24,42], when a steady state is assumed to have been reached. However, in a randomised controlled clinical trial, critically ill patients demonstrated suboptimal drug exposure. Both groups (400 mg twice daily vs. 200 mg 4 times a day) showed low C_min_ steady state plasma concentrations [43]. Posaconazole also produces moderate inhibition of CYP34A, so interactions should be considered when administering this drug. Common adverse reactions associated with posaconazole are nausea, diarrhea and vomiting [44], although hepatotoxicity, hypokalemia, rash and QTc prolongation have also been described [45,46].

**Table 2:** Summary of Clinical Studies Assessing PK Parameters of Posaconazole.

Clinical Context	Dose	AUC_0–24_ (mg h/L)	C_min_ (mg/L)	C_max_ (mg/L)	Cl (L/h)	Vd (L)	Reference
27 patients in the general intensive care unit	Oral suspension, 200 mg 4 times daily	0.217	0.137	0.084	-	-	[43]
27 patients in the general intensive care unit	Oral suspension, 400 mg twice daily	0.762	0.306	0.111	-	-	[43]
Subjects at high risk of invasive fungal disease (neutropenic patients receiving cytotoxic chemotherapy)	IV, 200 mg once daily	28.2	0.96	1.95			[47]

### 2.3. Voriconazole

Voriconazole is also a broad-spectrum antimycotic, with wide coverage of various microorganisms such as *Candida*, *Cryptococcus neoformans*, *Aspergillus* and others [22,24]. Like the other azoles discussed previously, voriconazole also has non-linear pharmacokinetics, which makes treatment more challenging [24]. For TDM, the main parameter used for voriconazole is serum concentration, in which levels >1 mg/L are associated with better clinical prognosis [24], while levels above 6 mg/L are considered to increase the risk of drug-related toxicity [23]. Plasma levels should be obtained on the fifth day after initiating antifungal treatment. Voriconazole requires special consideration regarding drug toxicity, since this drug has a high affinity for CYP2C19 [19], an enzyme with multiple polymorphisms in the general population, which might lead to increased susceptibility of developing adverse reactions [48]. Voriconazole toxicity is associated with liver and neurological damage, although cardiac abnormalities such as the development of arrhythmias as well as QTc prolongation have also been described.

Due to the concerns mentioned above, the importance of TDM with voriconazole has been studied profoundly in the last years. Park et al. found that TDM (maintaining plasma levels in the 1–5 mg/L range) significantly reduced the occurrence of adverse reactions and treatment discontinuation due to intolerance in patients treated for IFIs, or when voriconazole was used as empirical treatment [49]. Bienvenu et al. also evidenced an augmentation in the incidence of adverse reactions with higher plasma concentrations, as well as showing that higher SOFA scores (10 or higher) in critically ill patients were associated with higher voriconazole plasma concentration levels [50]. In general, the EUCAST (European Committee on Antimicrobial Susceptibility Testing) states that a C_min_ > 1–2 mg/L is associated with an approximately 70% response rate in adult patients. Table 3 summarizes the available evidence of PK parameters in clinical studies.

**Table 3:** Summary of Clinical Studies Assessing PK Parameters of Voriconazole.

Clinical Context	Dose	AUC_0–24_ (mg h/L)	C_min_ (mg/L)	C_max_ (mg/L)	Cl (L/h)	Vd (L)	Reference
Patients with venous haemofiltration	IV, 6 mg/kg twice daily on day 1; maintenance dose 4 mg/kg twice daily	44.8 ± 7.4	1.1 ± 0.3	5.9 ± 2.9	12.9 ± 6.7	2.96 ± 0.55	[51]
454 patients with invasive aspergillosis	IV, 6 mg/kg twice daily on day 1; maintenance dose 4 mg/kg twice daily	100.2 ± 43.08	3.10 (52)	-	-	-	[52]

### 2.4. Fluconazole and Isavuconazole

The last two azoles have not shown successful outcomes when performing TDM in critically ill patients [22]. Fluconazole is an azole with good coverage for most *Candida* species (except *C. krusei* and *C. glarbata*) [53]. Thanks to its linear pharmacokinetics as well as its renal excretion, requiring dosage adjustment only when there is severe renal function impairment, TDM might be recommended for dealing with CNS disease, or in patients in renal replacement therapy, and infection with an organism with a high MIC [24,54]. Nevertheless, adverse drug reactions are not rare with the usage of concomitant drugs, due to the high interaction between azoles and cytochrome P450 [55]. In these scenarios, reaching an AUC/MIC ratio close to 100 might lead to better outcomes [56].

Isavuconazole is the newest of the azoles, currently used for the treatment of invasive aspergillosis and mucormycosis [22]. Andes et al. argued against TDM in isavuconazole treatment due to the drug’s stable pharmacological properties and the absence of defined concentration thresholds [57], although they also stated that TDM might be warranted in patients who are being treated with the drug and dealing with therapeutic failure or unexplained liver injury, as well as obese patients, patients < 18 years old or those with a past medical history of moderate hepatic failure [58].

### 2.5. Recommendations for Azole TDM

As described earlier, TDM of azole therapy in critically ill patients can be beneficial not only in terms of giving a safer exposure (in addition to taking covariates like height, weight, liver/renal function, etc., into account), but also by decreasing the chances of therapy failure [32]. Nevertheless, due to the pharmacokinetics of certain drugs of this family, as well as to facilitate clinical practice and reduce costs, TDM in azoles should only be considered when using itraconazole, posaconazole or voriconazole as antifungal therapy.

## 3. TDM of Echinocandins in Critically Ill Patients

Echinocandins are a class of antifungal drugs targeting fungal cell wall synthesis. They act through non-competitive inhibition of the b-(1,3)-*D*-glucan synthase, the enzyme responsible for the synthesis of b-(1,3)-*D*-glucan, which is an essential component of the fungal cell wall. Although all echinocandins exhibit the same mechanism of action, they differ in certain PK properties. However, they all demonstrate extensive protein binding, lack of renal Cl, and lack of oral bioavailability [59,60]. The only fungi susceptible to this class of antifungals are *Candida* and *Aspergillus* spp. Their clinical use has increased significantly as they are recommended by some guidelines as the first-line therapy for candidemia, and for chronic disseminated candidiasis [14]. Echinocandins are also used as empiric therapy in patients with neutropenic fever, and in combination with triazoles as the initial treatment for invasive aspergillosis [33].

Due to the increased use of this class of antifungal drugs, resistance rates are increasing. For instance, Pfaller et al. documented an increase of 2.6% in the quantity of resistant candida isolates for Caspofungin and *C. parapsilosis,* 3.3% for micafungin and *C. Krusei,* and 1.4% for anidulafungin and *C. gablatra*, documented over a period of 9 years in 100 centres worldwide [13]. Echinocandin resistance can occur as a result of substitution mutations in the *FKS1* and *FKS2* genes. These amino acid substitutions lead to decreased sensitivity of the glucan synthase to the drug, and can elevate MIC values 5- to 100-fold [61]. The increasing resistance can be in part due to suboptimal drug exposure in critically ill patients, given that echinocandins demonstrate concentration- and dose-dependent efficacy [16]. There are several studies addressing the PK and PD changes of echinocandins in critically ill patients, some with conflicting results but the majority showing suboptimal exposure, as well as changes in Vd and Cl and changes in PK/PD indices compared to healthy volunteers [17,62,63,64].

However, there is no clear opinion on whether TDM is useful for echinocandins. An understanding of the relationship between antifungal exposure and response is necessary to adequately specify clinical thresholds that would have an impact on clinical outcomes and consequently the usefulness of TDM. In this section, we revise the available evidence to address the relationship between systemic echinocandin concentrations and efficacy, as well as inter-individual variability and covariates that may affect the drugs’ PK, before finally giving recommendations for TDM.

### 3.1. Anidulafungin

Anidulafungin is a semisynthetic water-insoluble compound extracted from fermentation products of the fungus *Aspergillus nidulans*. Anidulafungin differs from other echinocandins in that it is cleared from the body by non-enzymatic slow chemical degradation in the plasma; therefore, the drug has neither hepatic metabolism nor renal excretion, giving it a clinical advantage in that no dose adjustment is needed for hepatic or renal failure [65]. As with all echinocandins, anidulafungin has no oral bioavailability and is only administered by the intravenous route. It is a first-line treatment for candidemia, and it is given as an initial loading dose of 200 mg followed by 100 mg daily [14]. However, these doses were initially calculated for non-critically ill patients [66].

Preclinical evidence suggests anidulafungin exhibits a concentration-dependent killing pattern, and AUC/MIC and C_max_/MIC (ratio of the maximum serum drug concentration to the MIC) among the PK/PD indices have been the most predictive of in vivo efficacy [67]. In a neutropenic murine model of disseminated candidiasis, Andes et al. found that the anidulafungin doses required for fungal killing of 1 log CFU (colony forming unit) for various *candida* organisms (*C. albicans*, *C. glabrata*, *C. tropicalis*) were 2–13 mg/kg/24 h, and based on nonlinear regression analysis the C_max_/MIC and AUC/MIC were the most predictive parameters of treatment success [67]. Furthermore, given the increasing rates of azole-resistant invasive aspergillosis, echinocandins have emerged as an alternative chemotherapeutic option for *A. fumigatus.* Studies have evaluated the efficacy of of echinocandin dose escalation against *A. fumigatus*, in which anidulafungin needed an fAUC_0–24_/CLSI (Clinical & Laboratory Standards Institute) MEC (minimum effective concentration) of 766 to achieve 99.9% of the maximal activity [68].

According to EUCAST, the following parameters may be predictive for efficacy against *Candida* spp.: AUC_0–24 h_ 110 mg·h/L, C_max_ 7 mg/L, C_min_ 3 mg/L [69]. However, many clinical studies have detected suboptimal exposure of anidulafungin in critically ill patients. For instance, Liu et al. carried out an open label, multicentre study in 21 adult ICU patients with invasive candidiasis. The average AUC_0–24,_ and C_max_ were 92.7 mg h/L and 7.7 mg/L respectively, lower than in healthy volunteers [70]. Similar results have been obtained in many other studies, for example a clinical trial which evaluated 20 ICU patients with invasive candidiasis treated with the standard anidulafungin dose of 100 mg daily after a 200 mg loading dose. The mean AUC_0–24_ was 69.8 ± 24.1 mg·h/L, C_max_ was 4.7 ± 1.4 mg/L, and C_min_ was 2.2 ± 0.8 mg/L [62]. Suboptimal exposure was also documented in a prospective multicentre observational study of ICU patients with suspected intra-abdominal candidiasis, which found C_max_ of 6.0 ± 1.8 mg/L, C_min_ 3.2 ± 1.2 mg/L, and AUC_0–24_ 88.9 ±34.3 mg h/L [71]. Additionally, population PK data of anidulafungin obtained from 23 critically ill patients with invasive fungal infection showed a mean AUC_0–24_ of 102.19 mg h/L, with changes in clearance and therefore exposure in heavier patients with lower PTA [72]. On average, based on the available clinical evidence (Table 4), there was a decrease in the AUC_0–24_ of ≈23% in critically ill patients compared with healthy volunteers [15,62,71,72,73].

While these studies have generally shown suboptimal drug exposure, in some of them the concentrations were sufficient due to low MICs (<0.03 mg/L), and the required PTA was achieved. Some authors concluded that in overweight patients, or with candida isolates with higher MICs, a dose adjustment and TDM is warranted. Most recently, this phenomenon was evaluated in a systematic review and meta-analysis, where it was confirmed that critically ill patients treated with anidulafungin had lower AUC_0–24_ compared with healthy volunteers, as well as a lower C_max_ [64]. Table 4 summarises the available clinical studies regarding anidulafungin drug exposure in critically ill patients.

Given that echinocandins’ mechanism of action targets a component unique to the fungal cell wall, the relationship between systemic concentrations and toxicity is less relevant because significant side effects are uncommon. Humans lack the target that echinocandins aim at, so drug-related toxicity is not frequent. Infrequently reported serious side effects include modest elevation of transaminases (7–14%) and alkaline phosphatase, and infusion hypersensitivity reactions (rash, pruritus, bronchospasm) related to infusion velocity, while less severe side effects include pain at injection site and gastrointestinal complaints, among others [74]. Recently, a systematic review and meta-analysis that included 18,230 participants concluded that echinocandins are very safe and are the most tolerated antifungal [75].

Large inter-patient variability makes the relationship between the dose and the plasmatic concentrations not entirely reliable, so is one of the reasons for performing TDM. Great inter-individual and inter-occasion variability was found in a recently published anidulafungin PK model in critically ill patients, where inter-individual variability for Cl was 45.1%, for central volume 59.2%, and for peripheral volume 37%. AUC_0–24_, although decreased in the critically ill, does not seem to be the only factor contributing to reduced drug exposure. A high inter-individual variability in clearance can reduce drug exposure and can result in low PTA [17]. Inter-individual variability of anidulafungin was also documented in the DALI study, in which the coefficient of variation ranged between 28–57% in critically ill patients treated with anidulafungin or caspofungin [18].

Covariates have also been analysed to account for decreased echinocandin exposure in critically ill patients, for example body weight, body mass index (BMI), albumin levels, illness severity and age. There is conflicting evidence, because some studies found that gender, BMI, illness severity scores (APACHE II, SOFA etc.) and serum albumin did not influence the PK parameters of anidulafungin [15,62]. However, a relationship between higher SOFA scores and lower clearance values has been documented in other models [17]. Bodyweight has not been shown to have a significant correlation with anidulafungin exposure [62]; nonetheless, patients with a weight above 140 kg have been shown to have a mean AUC_0–24_ of <99 mg h/L, suggesting the need for a 25% increase in loading and maintenance doses for these patients [76].

### 3.2. Caspofungin

Caspofungin was the first echinocandin to be approved by the FDA. It is a semisynthetic lipopeptide derived from the fermentation product of the fungus *Glarea lozoyensis*. It is used as a first-line therapy for candidemia at a loading dose of 70 mg and a maintenance dose of 50 mg daily [14]. Caspofungin differs from anidulafungin because it is metabolised by hydrolysis and N-acetylation [59] and it is excreted in the urine and feces. Thus, the label recommendation for patients with liver impairment is a dose reduction, with 35 mg as a daily dose compared to 50 mg [77].

As with anidulafungin, caspofungin exhibits concentration-dependent reduction in fungal growth [78]. Thus, the proposed PK/PD indices related to efficacy in preclinical studies have been AUC_0–24_ and AUC/MIC [79,80]. Clinical evidence of the PK behavior of caspofungin in critically ill patients is conflicting. In the DALI study, the AUC_0–24_ was 52.0 mg h/L, the C_max_ was 3.9 mg/L, and the C_min_ was 1.5 mg/L, compared to the figures for healthy volunteers of 97.0 mg h/L, 12 mg/L, 1.4 mg/L respectively, suggesting suboptimal exposure of caspofungin in critically ill patients [18]. Furthermore, high inter-individual variability in Cl of ≈4.7% has been documented, and has been linked with a decrease of >20% in caspofungin exposure [81].Based on Cl calculations after 3 doses, the authors estimated that a maintenance dose of 50 mg would result in AUC_0–24_ of 89, 68 and 50 mg h/L for Cl values of 0.563 L/h, 0.737 L/h and 1.01 L/h respectively [81], none of which would be sufficient exposure when observing the minimum target required for efficacy (AUC_0–24_ 97 mgh/L) [82].

Suboptimal exposure was also seen in a multicenter prospective study covering 20 ICU patients with suspected invasive candidiasis, where the median caspofungin AUC_0–24_ was 78 mg·h/L, C_max_ 7.4 mg/L and C_min_ 1.7 mg/L. In 50% of the patients, dose adjustments had to be made because they had a AUC_0–24_ below 79 mg·h/L. Interestingly, the authors found that low caspofungin exposure was correlated with body weight above 75 kg and hypoalbuminemia [83]. Further studies have documented suboptimal exposure in critically ill patients with sepsis, for whom the mean AUC_0–24_ was 89 mg·h/L, C_max_ 10.5 mg/L and C_min_ 2.6 mg/L. A Monte Carlo simulation was performed and showed that PTA was achieved only for MICs < 0.03 mg/L when AUC_0–24_ was <75 mg·h/L; however, a PTA of ≈90% for a MIC of 0.125mg/L was not achieved. The authors concluded that in order to obtain the AUC seen in healthy volunteers, the median loading dose should have been 2.3 times higher than the standard loading dose. The changes were attributed to the septic state, where the PKs of hydrophilic compounds such as caspofungin are greatly altered [84]. Table 5 summarises the available clinical studies regarding caspofungin drug exposure in critically ill patients. On the other hand, a recently published systematic review and meta-analysis documented that when receiving caspofungin at a standard dose (70 mg loading dose, 50 mg maintenance), the C_max_ at a steady state and the AUC_0–24_ in critically ill patients were similar to healthy volunteers; however, it only took into account the measurements of a few studies [64].

**Table 5:** Summary of Clinical Studies Assessing PK Parameters of Caspofungin in Critically Ill Patients.

Clinical Context	Dose	AUC_0–24_ (mg h/L)	C_min_ (mg/L)	C_max_ (mg/L)	Cl (L/h)	Vd (L)	Reference
1. ICU patients with suspected or proven invasive candida infection	Standard *	57.8 (51.6 to 69.8)	-	-	0.88	11.9	[12]
2. ICU patients administered with caspofungin	Standard *	52.0	1.5	3.9	-	-	[18]
3. Critically ill adult patients with suspected or proven invasive candidiasis receiving continuous venovenous hemodiafiltration	Standard *	Arterial: 102 ± 46 Venous: 123 ± 46	2.4 ± 0.8	9.3 ± 2.3	0.630 ± 0.225	16.4 ± 5.4	[73]
4. ICU patients with suspected invasive candidiasis	Standard *	78 [IQR], 69 to 97 mg	1.7 (1.1–3.9)	7.4 (4.7–14.7)	0.66 (0.37–1.26)	9.1 (5.5–13.2)	[83]
5. ICU septic patients receiving caspofungin as empirical treatment	Standard *	89.2	2.6	10.5	0.06	9.3	[84]
6. ICU patients administered with caspofungin	Standard *	88.7 (72.2–97.5)	2.15 (1.40–2.48)	7.51 (6.05–8.17)	0.57 (0.54–0.77)	7.72 (6.12–9.01)	[85]
7. ICU patients with Child–Pugh B	70 mg loading dose followed by 35 mg daily	65 (22–241)	-	-	0.55	-	[86]
8. ICU patients receiving caspofungin	140 mg loading dose	79.1 (IQR 55.2; 108.4)	-	-	-	-	[87]

* Standard dose of caspofungin: loading dose of 70 mg and a maintenance dose of 50 mg daily.

As mentioned previously, a reduced dose of 35 mg daily compared to 50 mg is suggested for patients with liver impairment. However, this dose resulted in an AUC_0–24_ of 65 mg·h/L in critically ill patients, with a PTA of >99% for MICs of 0.03 mg/mL, but for MICs of 0.06 mg/mL it had a PTA of 81% for patients with a body weight of <80 kg, and a PTA of 59% for heavier patients. Thus, the authors concluded that the maintenance dose should not be reduced in non-cirrhotic patients if the Child–Pugh classification (used to assess the severity of liver disease depending on the degree of ascites, serum bilirubin and albumin concentrations, prothrombine time, and degree of encephalopathy) was driven by hypoalbuminemia [86]. Hypoalbuminemia has been another covariate analysed in relation to drug exposure in critically ill patients. Kurland et al. performed a study to assess the relationship between the PK of caspofungin in critically ill patients and liver function, plasma albumin levels and bilirubin levels. Most of the patients had a Child–Pugh B score, and the mean AUC_0–24_, Cl and Vd scores obtained can be seen in Table 5 [12]. The authors found no correlation between Child–Pugh scores and the AUC; however, there was a negative correlation between lower albumin levels and lower AUC, speculating that an increased free fraction of caspofungin would lead to a more effective elimination. However, this association with hypoalbuminemia is controversial and in other studies no link with drug exposure was shown [85]. The suggestion is not to reduce the maintenance dose in critically ill patients with abnormal function tests in the absence of chronic liver disease, but rather to maintain a daily dose of 50–70 mg based on the MIC values of the pathogen [12,86].

Other covariates have also been analysed to account for decreased caspofungin exposure in critically ill patients. For instance, body weight has been found to correlate with lower C_max_ and lower AUC_0–72_, regarding which the authors concluded that weight was directly correlated with Vd and Cl [88]. A more recent study compared a weight-based dose regimen, in which a loading dose of 2 mg/kg was followed by a maintenance dose of 1.25 mg/kg, with a standard regimen. With the standard regimen, the percentages of patients that achieved the target AUC (≈98 mg h/L) were 73%, 14% and 0% for patients of 50 kg, 78 kg and 120 kg respectively. For the weight-based regimen with the previously stated doses, the PTA was 98%, obtaining an AUC of ≈200 mg h/L. Toxicity was not a major concern given the safety of echinocandins [89]. This suggests a loading dose of approximately 140 mg may be appropriate for critically ill patients. Actually, a loading dose of 140 mg in critically ill patients was evaluated by Bailly et al. in a clinical trial (NCT02413892); however, they obtained an AUC_0–24 h_ of 79 mg h/L, which was similar to the concentrations observed after a loading dose of 70 mg. However, the patients included in that study were adult ICU patients with severe candidiasis, under mechanical ventilation, and receiving more than 0.1 μg/kg/min of epinephrine or norepinephrine, which were not inclusion criteria in the studies mentioned previously [87].

### 3.3. Micafungin

Micafungin is a water-soluble semisynthetic compound synthesised from the fermentation product of the fungus *Coleophoma empetri*. It is recommended for the treatment of candidemia, invasive candidiasis, and esophageal candidiasis, at a dose range of 50 to 150 mg daily with no recommendation for a loading dose [14,59]. It displays linear PK with daily doses of 50 mg to 150 mg and 3 mg/kg to 8 mg/kg body weight [90]. It has also been shown to be a mild inhibitor of CYP3A4. Micafungin is metabolized in the liver and approximately 90% of plasma clearance is through biliary elimination and fecal route [91]. In contrast to caspofungin, it does not require dose reduction in hepatic failure [59]. As with the other echinocandins, micafungin exhibits concentration-dependent killing but has also demonstrated dose-dependent efficacy in preclinical studies using models of neutropenic rabbits with disseminated candidiasis [92].

Critical illness has been associated with reduced micafungin exposure, demonstrated as a lower AUC_0–24 h_ after receiving standard doses in ICU patients compared to healthy volunteers (Table 6). Lower drug exposure represents a problem because the PK/PD clinical marker suggested for micafungin efficacy is the AUC/MIC ratio [93]. For instance, neutropenic murine models of disseminated candidiasis have shown that the total AUC/MIC ratio required to inhibit growth of *C. albicans* is 2420, and for *C. glabrata* 1283; however, higher drug exposure is required for fungicidal activity. Thus, based on clinical evidence, the target AUC/MIC ratios are >3000, >5000 and ≥285 for *Candida* spp., Non-*C. parapsilosis* and *C. parapsilosis*, respectively [94]. A prospective PK study in ICU patients treated with micafungin for a suspected or proven invasive candida infection documented a mean AUC_0–24 h_ of 89.6 mg h/L while using a standard dose of 100 mg daily. In 17.6% of the patients the target AUC/MIC [95] was not met. These levels were below the AUC_0–24 h_ found in healthy volunteers: 152.0 mg h/L and 134.0 mg h/L [96,97]. Furthermore, the authors found a correlation between bodyweight and decreased micafungin exposure, where patients with a body surface area of >2.10 m^2^ or a fat-free mass of >62 kg had significantly lower drug exposure. No correlation was found between drug exposure and albumin levels, nor with liver function tests, C-reactive protein or total bilirubin [95].

Another study evaluated micafungin exposure in ICU patients with sepsis and mechanical ventilation. They found that albumin levels and SOFA scores were associated with drug exposure, where the AUC varied according to these parameters. Accordingly, the AUC was 87.3 mg h/L (SOFA score ≥ 10 and albumin levels ≤ 25 g/L), 65.5 mg h/L (SOFA score < 10 and albumin levels ≤ 25 g/L), 99.5 mg h/L (SOFA score ≥10 and albumin levels > 25 g/L) and 74.6 mg h/L (SOFA score < 10, and albumin levels > 25 g/L) [98]. As with other echinocandins, micafungin has high protein binding (99%), thus a hypothesis for low drug exposure in the critically ill is that hypoalbuminemia results in increased free drug and increased total clearance. Weight is another covariate that must be taken into account for the altered PK of micafungin. Maseda et al. performed a PK model including critically ill non-obese and critically ill morbidly obese patients, to estimate if they reached the target AUC/MIC. Micafungin exposure was adequate with doses of 150 mg daily for patients with weights up to 115 kg, and with 200 mg daily for patients weighing >115 kg, however, regardless of weight or *Candida* spp., 100 mg daily did not lead to adequate drug exposure [99]. In addition, in addition to the documented low drug exposure of micafungin at steady states, there is also evidence suggesting that on the first day of treatment the PTA is lower than 80%, hinting at the need for a loading dose. Kapralos et al. showed that a loading dose of 200 mg or even 300 mg could improve efficacy and increase the PTA [100].

**Table 6:** Summary of Clinical Studies Assessing PK Parameters of Micafungin in Critically Ill Patients.

Clinical Context	Dose	AUC_0–24_ (mg h/L)	AUC/MIC ratio	Cl (L/h)	Vd (L)	Reference
1. ICU patients receiving micafungin for suspected or proven fungal infection	Standard *	91 (67–122)	-	1.10	17.6	[86]
	150 mg daily	137 (101–183)	-			
	200 mg daily	183 (135–244)	-			
2. ICU patients treated with micafungin for a suspected or proven invasive candida infection	Standard *	89.6	6221 (*C. albicans*) 5643 (*C. glabrata*)	-	-	[95]
3. ICU patients with sepsis and mechanical ventilation	Standard *	65.5 **	-	1.34 L/h	11.80	[98]
4. ICU patients treated with micafungin for a suspected or proven invasive candida infection	Standard *	76.33	-	1.31 L/h	14.2 L	[100]

* Standard dose for micafungin: 100 mg daily ** In patients with a SOFA score < 10, and albumin levels ≤ 25 g/L.

### 3.4. Recommendations for Echinocandin TDM

Finally, even though echinocandins have been demonstrated to be safe and to have very little toxicity at high doses that could justify TDM, exposure monitoring should be considered in patients with variable PK, including critically ill patients. TDM of echinocandins in critically ill patients would lead to dose adjustment to guarantee target concentration or exposure, which in the case of echinocandins is closely related to efficacy, as previously discussed. To estimate drug exposure (AUC), multiple drug concentrations must be obtained over a single dosing interval, but this is not practical. Thus, a limited sampling strategy is the proposed method to estimate drug exposure. For instance, Van Wanrooy et al. documented that anidulafungin exposure could adequately be estimated using the concentration from a simple sample taken 12 h after the start of the infusion using linear regression analysis or a population PK model [101]. This dosing software allows complex PK/PD parameters to be calculated, and includes linear-regression-based dosing software, population PK-based dosing software, and Bayesian forecasting dosing software [102]. We believe that AUC-guided echinocandin dosing and monitoring for critically ill adult patients could improve clinical outcomes in the ICU.

## 4. TDM of Amphotericin B in Critically Ill Patients

Amphotericin B, amphotericin A and nystatin comprise the family of polyene antifungals. Discovered in 1956 [103] and initially approved in 1959, amphotericin B is the central drug of the polyenes [104] and although new antifungal agents (from different families) have recently been introduced in clinical practice, it remains the mainstay therapy for many severe and opportunistic fungal infections.

Its molecular structure comprises a 38-member macrocyclic ring with four main domains: (1) a 14-carbon hydrophobic polyene chain, (2) a polyhydroxylated carbon chain with seven free hydroxyl groups, (3) a hydrophobic tail with one free hydroxyl group, and (4) a polar head containing a mycosamine residue and side chain with a free amino group [105,106]. Despite possessing amphipathic properties, amphotericin B is primarily insoluble in water, and thus requires the addition of excipients in order to gain stability and exert its desired clinical effects. The first described formulation was with sodium deoxycholate, a compound that increases amphotericin B’s solubility and its ability to create stable micellar suspensions [106,107]. While sodium deoxycholate initially appeared to be the appropriate solution to ensure structural stability, toxicity-related side effects (discussed below) emerged in clinical use, which led to the development of safer forms, namely lipid-enhanced formulations including liposomes, emulsions and lipid complexes, some of which can be seen in current practice [108,109,110,111]. Moreover, current research has also started to include formulations of amphotericin B in lipid-polymer hybrids (nanoemulsions) and specialised micelle nanocarriers [112,113].

The main mechanism of action of amphotericin B (and polyenes in general) relies on the binding with membrane sterols, predominantly ergosterol, and the subsequent disruption of the cell-medium electrolytic concentration homeostasis–particularly that of K^+^, Mg^2+^ and Ca^2+^–leading to cell death [114,115]. While not completely understood, research has also suggested an ergosterol-independent mechanism through which amphotericin B, in considerably higher concentrations, can form membrane pores that result in fungal death [116,117]. Additionally, a second, immune-mediated mechanism of action through which amphotericin B exerts antifungal effects has been described. Studies have demonstrated that amphotericin B induces oxidative stress in cells through the expression of stress genes, increases the inducible form of nitric oxide synthase, and increases the induction of proinflammatory cytokines [118,119,120]. Interestingly, the mechanism of action has been shown to cause in vitro damage to *Trypanosoma cruzi* and *Leishmania* spp. [120]. Moreover, the drug is known to interact with immune-specific cell receptors such as toll-like receptors 2 and 4, respectively causing an increase in proinflammatory and anti-inflammatory cytokine production [121,122,123,124].

As previously mentioned, amphotericin B is known to have adverse effects that range from an innocuous rash following initial infusion, up to acute kidney injury that, although uncommon, may even warrant renal replacement therapy [125]. Adverse effects associated with amphotericin B vary according to the formulation used. They are known to occur more frequently with sodium deoxycholate as the main excipient, and less frequently with lipid-based formulations [126]. Moreover, regardless of the mechanism that causes them, side effects can be classified as either (a) infusion-associated adverse effects, or (b) direct toxicity caused by amphotericin B. The first group most commonly present as immune-mediated symptoms caused in the majority of cases by the interaction of the formulation excipients with innate immune response mechanisms; these effects may include skin rashes, allergic rhinitis, generalised pruritus and (rarely) anaphylaxis [126]; most of these side effects can be prevented or treated with a premedication regime including acetaminophen with or without antihistamine agents (e.g., diphenhydramine) [127]. The latter group, direct toxicity caused by amphotericin B, are hypothesised to be caused by interaction of amphotericin B with membrane cholesterol molecules. These are the most severe reactions and include nephrotoxicity, often presenting as a reversible elevation of the basal levels of creatinine; electrolyte disturbances including hypokalemia, hypomagnesemia and hyperchloremic metabolic acidosis; and hematologic abnormalities such as anemia, leukopenia and, less frequently, thrombocytopenia [126,128,129,130].

Regarding its therapeutic spectrum, amphotericin B possesses arguably the broadest coverage amongst all antifungal agents. Although some conflicting evidence exists for certain species, there is consistency regarding most of the species considered both susceptible and resistant to amphotericin B. Amongst susceptible organisms, *Candida tropicalis*, *Candida krusei*, *Candida guilliermondii*, *Candida kefyr* and *Candida famata* stand out from the Candida genus, while *Malassezia* spp., *Saccharomyces cerevisiae*, *Aspergillus niger*, *Aspergillus nidulans* and *Cryptococcus neoformans* are also considered sensitive [131,132,133]. Compellingly, *Candida albicans* and *Candida parapsilosis*, while still believed to be susceptible, have been reported to exhibit resistance [132,134,135,136]. Regarding resistant species, *Candida auris*, *Candida lusitaniae*, *Candida haemulonii*, *Sporothrix schenckii* and *Scedosporium prolificans* are the most noteworthy organisms known to cause disease in human hosts [137,138,139].

*Aspergillus fumigatus*, *Aspergillus flavus*, *Fusarim* spp., *Trichosporon beigelii* and *Scedosporium apiospermum* are some of the organisms that have generated controversial results regarding amphotericin B resistance and clinical utility in spite of reports of therapeutic failure [138,140]. Lastly, it should be mentioned that difficulty in culturing and testing antifungal resistance in Zygomycetes makes it difficult to obtain a clear point of reference regarding their susceptibility; nonetheless, amphotericin B remains a useful tool in severe *Mucor* spp., *Rhizopus* spp., and *Rhizomucor pusillus* infections [141].

Invasive mycoses are a common complication in critically ill patients, a population characterised by a profound comorbidity burden, and in many cases, variable states of immune deficit [142,143]. Under these circumstances, treatment of fungal infection requires a precise and fine-tuned approach in order to reach optimal outcomes. TDM has recently emerged as a practice that may provide better results by optimising–dosages and administration intervals through individualised approximation, maintaining adequate therapeutic concentrations and decreasing the risk of toxicity-related adverse effects [22].

### Recommendations for Amphotericin B TDM

In the critically ill, there are several considerations that have an impact in drug concentrations over time. For instance, age, sex, comorbidities, current condition, renal function, and other concomitant drugs are known factors to consider [144]. Currently, among antifungal agents, amphotericin B is not routinely monitored through TDM [24,32,144]. This is mainly because, as previously mentioned, the PK and PD characteristics of amphotericin B (despite over 60 years of clinical practice experience), are not completely understood, and vary with each different formulation [145]. In that regard, it is not desirable routinely to use TDM with amphotericin B, except in special scenarios in which a narrow therapeutic range is desired and/or toxicity is a major concern.

## 5. Flucytosine

The pyrimidine analogue flucytosine was initially synthesised in 1957 as a chemotherapeutic agent with anti-tumour properties. However, it proved inferior to other available drugs and quickly became overshadowed [146,147]. Some years later, in vitro studies showed that flucytosine had previously unknown antimicrobial properties, which revived clinical and experimental interest in the drug [148,149,150]. While flucytosine has no intrinsic antifungal effects, its metabolite 5-fluorouracil is known to inhibit both RNA and DNA synthesis in fungal cells. This process mainly occurs through the deamination of flucytosine by cytosine deaminase in fungal cell cytoplasm shortly after it is taken up by cytosine permease [151]. It should be noted that 5-fluorouracil is highly toxic to human and non-human mammalian cells and is not readily taken up by fungal cells, thus leaving it ineffective as an antifungal agent; instead, its main current use concerns the treatment of certain types of cancer, mainly colorectal carcinoma [152,153].

Flucytosine is a fluorinated analogue of cytosine, hence its interactions with cytosine permease and cytosine deaminase. It shares metabolic pathways with adenine, hypoxanthine and cytosine. Moreover, 5-fluorouracil–its active metabolite–exerts antifungal effects through two main mechanisms: (1) it accepts three phosphate groups and becomes 5-fluorouridine triphosphate, before being incorporated into fungal RNA in place of uridylic acid, altering tRNA aminoacylation and damaging protein synthesis, and (2) it is converted into 5-fluorodeoxyuridine monophosphate by uridine monophosphate pyrophosphorylase, becoming an inhibitor of thymidylate synthetase (a mechanism of action shared with the chemotherapeutic agent capecitabine), and disrupting the synthesis of new DNA molecules [154].

Flucytosine is active against most yeasts, including *Cryptococcus*, *Candida* and *Torulopsis* spp. Additionally, other types of fungi, such as *Aspergillus*, *Cladosporium*, *Phialophora*, *Cephalosporium*, *Sporothrix* and *Blastomyces* are sensitive to flucytosine [154,155]. Minimal inhibitory concentrations (MIC), while not thoroughly documented, have ranged widely from <100 mg/L up to over 1000 mg/L [155]. However, flucytosine resistance has been thoroughly described in clinical and laboratory conditions and is a matter of concern, making it a candidate for clinical follow-up through TDM. Importantly, intrinsic resistance has been reported in several species such as *C. albicans*, *C. neoformans* and *Torulopsis glabrata* in up to 7–8% of strains, yet different factors mean these rates vary geographically [156]. Resistance has been identified through two main mechanisms: (1) mutations in the enzymes necessary for the biological processes undergone by flucytosine that decrease its final effects [157,158], and (2) an upregulation of intrinsic pyrimidine production, which then leads to a competitive inhibition of flucytosine’s metabolites, reducing its antifungal effects [158]. Moreover, two flucytosine resistance phenotypes have recently been recognised [158,159,160]. Phenotype 1 is characterised by not being affected by flucytosine at high concentrations, and these fungi are therefore considered to have full resistance to flucytosine and its metabolites. On the other hand, phenotype 2 fungi are characterised by being susceptible to flucytosine, but they develop resistance after being exposed for a certain period, and are thus considered to have an inducible resistance to flucytosine.

Pharmacokinetically, while flucytosine’s dynamics have been extensively studied, certain doubts remain. Regarding absorption, there is controversy about where flucytosine is absorbed yet it possesses an oral bioavailability of 75–90% [161,162]. Flucytosine has high body fluid penetration, reaching important levels in peritoneal, synovial and cerebrospinal fluids [161,162,163,164,165,166]. Latency is usually short, with peak serum concentrations being readily reached in about 1–2 h. Due to its molecular structure, flucytosine does not bind strongly to serum proteins and is highly water-soluble [161,162,163,164,165,166,167]. Clearance and elimination mainly take place in the kidneys, and almost no liver metabolism has been described [151,161,164]. Moreover, it should be noted that neither tubular reabsorption nor secretion has been documented. Flucytosine’s half-life in healthy volunteers ranges from 3 to 4 h, but its excretion is in close relation to creatinine clearance, and can reach 70 to 86 h; thus, dosage adjustments are needed in patients with glomerular filtration rates below 40 mL/min [161,162,164].

Therapeutic monitoring has provided relatively scant evidence regarding flucytosine; nonetheless, due to several factors, TDM may prove a valuable tool for improving patient outcomes. Namely, high variability in serum concentrations following administration, lack of supporting clinical data on optimal concentrations for treatment, and severe adverse effects when concentrations exceed the toxicity threshold make flucytosine a candidate for TDM [168,169,170,171]. A summary of the PK parameters of flucytosine in clinical studies can be seen in Table 7. The main preoccupation that warrants a tailored dosing regimen for flucytosine is toxicity prevention. The most severe reactions include hepatotoxicity, bone marrow suppression and gastrointestinal disturbances [172,173,174,175]. These findings have been mostly associated with serum concentrations over 100 mg/L [172,176]. Hepatotoxicity has been reported in up to 25% of patients in some case series, and is mostly seen as ALT (alanine transaminase) and AST (aspartate aminotransferase) as well as alkaline phosphatase elevations [151,166,177]. Less commonly, hyperbilirubinemia and hepatomegaly can also occur [151,177]. Fortunately, this liver damage side effect can be easily reversed by decreasing dosages regardless of serum concentrations [172,178]. Gastrointestinal side effects occur in less than 10% of patients, and usually include vomiting, diarrhea, bloating and abdominal pain; as with liver toxicity, these side effects usually subside with decreased dosages [174,175]. Bone marrow suppression is the most severe side effect associated with flucytosine toxicity [179]. Available evidence suggests a concentration-dependent effect, with risk being present above 100 mg/L and critical in concentrations over 125 mg/L [177,178,179,180,181,182]. Leucopenia and thrombocytopenia are the most common findings, yet pancytopenia has also been reported in several studies [183,184]. Controversy remains whether HIV infection is a factor associated with higher incidence rates of myelosuppression [185].

**Table 7:** Summary of Clinical Studies Assessing PK Parameters of Flucytosine in Healthy Volunteers and Patients with Cryptococcal Infections.

Clinical Context	Dose	AUC_0–24_ (mcg h/mL)	C_min_ (mcg/mL)	C_max_ (mcg/mL)	Cl (L/h)	Vd (L)	Reference
Healthy volunteers given immediate-release oral flucytosine	13.8–25.4 mg/kg PO every 12 h	435	5.32	57.3	0.49	5.62	[186]
Healthy volunteers given controlled-release oral flucytosine (Methocel K4M)	13.8–25.4 mg/kg PO every 12 h	221	3.54	28.4	0.75	7.37	[186]
Healthy volunteers given controlled-release oral flucytosine (Methocel K100M)	13.8–25.4 mg/kg PO every 12 h	222	5.31	23.5	0.82	15.0	[186]
Case report of a patient with a severe cryptococcal infection undergoing CVVHDF	25.8 mg/kg PO every 12 h	2980	2.0	>8.0	1.92–2.31	48.1–57.7	[187]
Case report of a patient with a severe cryptococcal infection undergoing CVVHDF	25 mg/kg PO every 12 h	-	45	62–64	1.32–1.36	0.42–0.42 (per Kg)	[188]
Empiric antifungal coverage in a patient with refractory septic shock and requiring CVVHD	37.5 mg/kg on day 1 and 50 mg/kg after	-	34	110	1.1	57.4	[189]

### Recommendations for Flucytosine TDM

Current clinical recommendations suggest routine TDM for flucytosine, in light of the aforementioned reasons [144]. The recommended serum concentration range is 25–100 mg/L with an initial dosing regimen of 37.5 mg/kg every 6 h adjusted regardless of clinical indication and measured serum concentrations, which should be drawn ideally within the first 72 h, and no later than 120 h after initiation [24,190,191]. An approach suggested by the IDSA in its 2010 Cryptococcal Infections Guidelines is to target a 2 h post-dose concentration of >30 mg/L measured at least 72 h after treatment initiation [191]. Consensus exists in aiming for serum concentrations above 20–25 mg/L in order to diminish resistance induction rates, as previously mentioned, and below 100 mg/L to decrease toxicity as well as individually to tailor the lowest permitted concentration in regard to MIC.

## 6. Conclusions

In conclusion, antifungal drugs are widely used in critically ill patients. Critical states can alter the PK parameters of antifungal drugs to the point of lowering the probability of treatment success and contributing to antimicrobial resistance. In general, we consider that clinicians should adopt PK/PD-based dosing as part of routine clinical practice, in order to avoid the suboptimal exposure seen with the use of standard dosing. Due to the high toxicity of azoles and the high probability of drug-drug interactions, TDM should be considered when using itraconazole, posaconazole or voriconazole as antifungal therapy. For its part, echinocandin exposure has been shown to provide suboptimal exposure in critically ill patients and also in overweight patients, with documented high inter-individual variability, reasons that favor TDM. PK parameters are not well elucidated for amphotericin B so routine TDM is not desirable, except when toxicity is a major concern, or a narrow therapeutic range is needed. Finally, routine TDM for flucytosine is recommended due to the high variability in serum concentrations following administration and severe adverse effects when concentrations exceed the toxicity threshold. Furthermore, we consider further studies are needed to evaluate PK parameters during antifungal treatment in critically ill patients, especially those receiving amphotericin B, for which most studies to date have been conducted in immunocompromised pediatric patients.

## Figures and Tables

**Table 4:** Summary of Clinical Studies Assessing PK Parameters of Anidulafungin in Critically Ill Patients.

Clinical Context	Dose	AUC_0–24_ (mg h/L)	C_min_ (mg/L)	C_max_ (mg/L)	Cl (L/h)	Vd (L)	Reference
Critically ill patients with proven or suspected invasive fungal infection	Standard *	- Sample on day 3: 72.1 (IQR 61.3–94.0) - Sample on day 7: 82.7 (IQR 73.0–129.5	- Sample on day 3: 2.2 (IQR 1.9–2.9) - Sample on day 7: 2.8 (IQR 2.2–4.2)	- Sample on day 3: 5.3 (IQR 4.1–6.0) - Sample on day 7: 5.9 (IQR 4.6–8.0)	- Sample on day 3: 1.4 (IQR 1.1–1.6) - Sample on day 7: CL of 1.2 (IQR 0.8–1.4)	- Sample on day 3: 46.0 (IQR 32.2–60.2) - Sample on day 7: 39.7 (IQR 32.2–54.4)	[15]
ICU patients administered with anidulafungin	Standard *	114 ± 40.78	3.21 ± 1.43	9.27 ± 2.76	0.842	-	[17]
ICU patients administered with anidulafungin	Standard *	55	1.8	55	-	-	[18]
ICU patients with invasive candidiasis	Standard *	69.8 ± 24.1	2.2 ± 0.8	4.7 ± 1.4	1.6 ± 0.6	-	[62]
ICU patients with invasive candidiasis	Standard *	92.7	3.0	7.7	1.3	38.8	[70]
ICU patients with suspected intra-abdominal candidiasis	Standard *	88.9 ± 34.3	3.2 ± 1.2	6.0 ± 1.8	1.2 ± 0.5	72.8 ± 63.9	[71]
ICU patients with proven or suspected invasive fungal infection	Standard *	102.19	-	-	-	-	[72]
Critically ill patients with continuous venovenous haemodiafiltration	Standard *	93.9 ± 19.4 (arterial sample), 104.1 ± 20.3mg·h/L (venous sample)	3.0 ± 0.6	6.2 ± 1.7 (arterial sample) 7.1 ± 1.9 (venous sample)	-	-	[73]

* Standard dose: 200 mg loading dose followed by 100 mg daily.

## Data Availability

Not applicable.
